# *In vitro* Studies of the Antibody Response: Antibodies of Different Specificity are Made in Different Populations of Cells

**DOI:** 10.3389/fimmu.2014.00515

**Published:** 2014-10-28

**Authors:** Richard W. Dutton

**Affiliations:** ^1^Pathology Department, University of Massachusetts Medical School, Worcester, MA, USA

**Keywords:** *in vitro* culture, lymphocyte proliferation, clonal selection

Our paper (1) “Cell populations and cell proliferation in the *in vitro* response of normal mouse spleen to heterologous erythrocytes. Analysis by the hot pulse technique,” investigated the kinetics of antigen driven proliferation of antibody forming cells in culture and showed that different populations of cells were involved in the response to two different antigens, providing evidence for the clonal selection theory proposed by both Sir McFarlane Burnet and by David Talmage.

I was trained as a biochemist in England but switched to immunology when I was given the opportunity to come to the United States to join John Vaughan’s lab at the Medical College of Virginia in Richmond, Virginia. I arrived in January 1957 and was soon immersed in the great debates then raging in immunology.

At that time, the revolution in biology was well underway. The double helix model for DNA was propounded in 1953 but it took some time for the significance of the double helix to sink in. The “central dogma that DNA codes for RNA that codes for protein” came later and the nature of the genetic code and the mechanisms of protein synthesis (messenger RNA, transfer RNA, and ribosomes) were not completely worked out until 1964–66, more than 10 years later.

Immunology, however, was a separate arena. In 1957, most experiments in immunology were carried out in whole animals. Antigen in, wait 10 days, antibody out, and everything in between took place in a black box. The structure of the antibody molecule, immunoglobulin genes, cytokines, signaling pathways, and almost all of what we now study was all unknown.

It was thought that the architecture of the lymphoid organs was essential to their proper function (and indeed, we are just now coming back to that same understanding) and it worried people that half the spleen cells put out into culture were dead in 24 h. As a biochemist, accustomed to the study of metabolism in cell free fractions, I was not deterred and pushed ahead regardless. Our first goal was to get immune responses from single cell suspensions *in vitro* where we could study them and manipulate them under various conditions.

At that time, there was no *in vitro* system that did anything other than just demonstrate that antibody was being made and most employed tissue slices or fragments rather than single cell suspensions (2). The trick that led to our success was to start the response *in vivo* and then switch to culture and continue the response *in vitro*. The assay for antibody was the measurement of incorporation of radiolabeled amino acid into antibody that could be recovered by coprecipitation with antigen antibody complexes (2). Using this, we could quantitate the rate of antibody synthesis and measure the kinetics of the response and determine how it was affected by culture conditions. Our first application was to investigate the metabolic activity of antibody formation and to show that the incorporation radiolabeled phosphate into acid soluble, fat soluble, RNA, and DNA phosphate was increased in antibody forming cultures. From today’s perspective, this was not the most obvious thing to do but many investigators were conducting similar analyses in the study of protein synthesis. People looked on antibody synthesis in lymphoid organs in the same way that they looked on albumin synthesis in the liver. The protein was assumed to be made in all the cells of the organ, not as the product of small but rapidly expanding subset of the cells, and it seemed that the global analysis of the accompanying biochemical events might be revealing. This proved not to be the case but our focus was soon switched to more profitable studies.

We went that April, to the Symposium on “Antibodies: Their production and mechanism of action” sponsored by the Biology Division of the Oak Ridge National Laboratory, Oak Ridge, Tennessee. Presentations from the meeting were published in the Journal of Cellular and Comparative Physiology Volume 50, Supplement 1, December 1957 and contained papers by Frank Dixon, Jon Singer, Elvin Kabat, David Talmage, and N.A. (Av) Mitchison, to mention just a few that may still be known to the “elders” in our field. Also, presented were two papers by “young Turks,” one by Novelli and DeMoss (3) and the other by Schweet and Owen (4), both of which sought to apply the new understanding of the molecular biology of the control of protein synthesis to the synthesis of antibody. They received only a mixed reception from the old school immunologists, brought up in a discipline still isolated from the main body of biology.

After the meeting, we made our way by car to the annual AAI/FASEB meeting that year in Chicago but bumped into Talmage and his family in the caverns at Mammoth Caves in Kentucky, which led to an invitation to visit him in his house while at the meeting in Chicago. There, in the crowded kitchen, Talmage, Burnet, Vaughan, and others engaged in a vigorous argument of the pros and cons of the, not yet quite crystallized, clonal selection theory (5, 6), that stated the individual antibody forming cells were committed to the synthesis of just one unique antibody. The theory allowed one to explain many aspects of the induction of immunological tolerance and generated great excitement at the time.

In the years that followed, the clonal selection theory became generally accepted, based more on its intellectual appeal rather than on experimental evidence, which was actually somewhat conflicting.

Our contribution (1) in support of the theory was to show that cells making one antibody could be destroyed using a “hot pulse” technique without affecting a second population in the same culture that were making another antibody.

The lead-up to this began years earlier when we showed, in 1958, that an *in vitro* antibody response could be drastically reduced by 8-azaguaninne, an inhibitor of RNA and DNA synthesis (7). Burnet had suggested that the induction of antibody synthesis might be analogous to the induction of inducible enzymes (8) and Creaser had shown that inducible enzyme synthesis in bacteria could be strongly inhibited by 8-azaguaninne (9). We were worried, however, that the 8-azaguaninne appeared to be more generally toxic but our later studies showed that antibody formation was selectively inhibited by inhibitors of DNA synthesis while the synthesis of other proteins was less affected, suggesting that antibody synthesis was somehow dependent on DNA synthesis (10). We were able to confirm that the antibody forming cells were dividing (11) using an early version of the hot pulse technique and, in 1962, we showed that antigen actually stimulated DNA synthesis as measured by the increased uptake of tritiated thymidine in cultures of lymphocytes from previously immunized rabbits (12). It is, perhaps hard to believe from our current perspective, that this was a novel, exciting finding, but the “obvious” is often not “obvious” until it is “obvious.”

At that time, we did not know about T-cells and B-cells and the role of T-cells in the B-cell response, and we assumed that the dividing cells were the antibody forming cells. Now, we would presume that T-cells are also a component and the assay soon became a major assay in the hands of Benacceraf, and others, for many T-cell studies (13).

Later, in 1966, Mishell and I developed a more sophisticated *in vitro* model (14) in which we could generate a primary antibody response of mouse spleen cells to various erythrocyte antigens and it was this that we used to show that responses to different antigens were carried out by different cells. By this time, we had adopted the use of the hemolytic plaque assay (15), developed by Jerne and Nordin (16), and we could measure the actual number of cells making antibodies to erythrocyte antigens. We started the response to antigen A, killed the cells making the response by letting the dividing cells incorporate highly radioactive tritiated thymidine, diluted the tritiated thymidine with unlabeled thymidine, and then started the response to antigen B. The two antigens were sheep erythrocytes and burro (donkey) erythrocytes, and the results were the same whether we started with burro or sheep. Other experiments in the paper showed that the first round of proliferation did not begin until 24 h after the addition of antigen to naïve cells but was earlier if the cells were from immunized donors and that virtually all the antibody forming cells arose from the extensive proliferation of a much smaller number of precursor cells.

The idea for the hot pulse technique, which we used here and in our earlier paper (11), came from my undergraduate days where I had learned of the studies of Hershey et al. (17) in which they showed that phage infection of *Escherichia coli* could be progressively destroyed if the DNA was labeled with radioactive 32P. In their technique, it was the disintegration of the 32P that destroyed the link between successive nucleotide triphosphates of the DNA, while in our technique (11), we showed that it was the very soft beta irradiation of the incorporated tritium that killed only the cells that had incorporated the hot thymidine as they synthesized new DNA.

Subsequent studies with the same *in vitro* system led to the identification of a T-cell replacing factor (18), the effect of mitogens on T-cell help (19), positive and negative allogeneic effects (20), and the true nature of the relationship between CD8 and CD4 T-cells and the recognition of Class I and Class II MHC (21, 22).

In my memory, I had seen us led inexorably to the truth by a series of searing insights but as I reread the papers I see that we only stumbled our way to a better understanding.

## Conflict of Interest Statement

The author declares that the research was conducted in the absence of any commercial or financial relationships that could be construed as a potential conflict of interest.
